# Interlayer Interactions in 1D Van der Waals Moiré Superlattices

**DOI:** 10.1002/advs.202103460

**Published:** 2021-11-28

**Authors:** Sihan Zhao, Ryo Kitaura, Pilkyung Moon, Mikito Koshino, Feng Wang

**Affiliations:** ^1^ Interdisciplinary Center for Quantum Information Zhejiang Province Key Laboratory of Quantum Technology and Device State Key Laboratory of Silicon Materials Department of Physics Zhejiang University Hangzhou 310027 China; ^2^ Department of Chemistry Nagoya University Nagoya 464‐8602 Japan; ^3^ Arts and Sciences NYU Shanghai Shanghai 200122 China; ^4^ NYU‐ECNU Institute of Physics at NYU Shanghai Shanghai 200062 China; ^5^ Department of Physics Osaka University Toyonaka 560‐0043 Japan; ^6^ Department of Physics University of California at Berkeley Berkeley CA 94720 USA; ^7^ Materials Science Division Lawrence Berkeley National Laboratory Berkeley CA USA; ^8^ Kavli Energy NanoSciences Institute at University of California Berkeley and Lawrence Berkeley National Laboratory Berkeley CA 94720 USA

**Keywords:** moiré superlattices, 1D moiré superlattices, van der Waals heterostructures, double‐walled carbon nanotubes, interlayer interactions

## Abstract

Studying two‐dimensional (2D) van der Waals (vdW) moiré superlattices and their interlayer interactions have received surging attention after recent discoveries of many new phases of matter that are highly tunable. Different atomistic registry between layers forming the inner and outer nanotubes can also form one‐dimensional (1D) vdW moiré superlattices. In this review, experimental observations and theoretical perspectives related to interlayer interactions in 1D vdW moiré superlattices are summarized. The discussion focuses on double‐walled carbon nanotubes (DWNTs), a model 1D vdW moiré system, and the authors highlight the new optical features emerging from the non‐trivial strong interlayer coupling effect and the unique physics in 1D DWNTs. Future directions and questions in probing the intriguing physical phenomena in 1D vdW moiré superlattices such as, correlated physics in different 1D moiré systems beyond DWNTs are proposed and discussed.

## Graphical abstract

The progress on studying the interlayer interactions in one‐dimensional (1D) van der Waals (vdW) moiré superlattices is reviewed, with highlighting the unique physics of 1D moiré systems. Important questions and future directions in this emerging field are also discussed in this perspective.

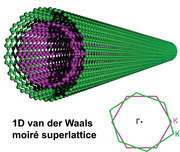

## 1 Introduction

Van der Waals (vdW) materials represent a large number of natural and artificial layered materials in which the stacking layers are weakly coupled by the vdW force, yet the atoms within each layer are ionically or covalently bonded.^[^ ^1^, ^2^ ^]^ Owing to the weak bonds between the layers, most of vdW materials can be exfoliated down to atomically‐thin layers.^[^ ^3^, ^4^, ^5^, ^6^, ^7^ ^]^ Remarkably, the vdW interlayer interactions in layered materials with different stacking orders can lead to profound differences in their electronic properties. For example, Bernal‐stacked graphite^[^ ^8^, ^9^, ^10^ ^]^ and rhombohedral graphite,^[^ ^11^, ^12^, ^13^ ^]^ two stable forms of crystalline graphite, exhibit very different electronic properties. When graphite is thinned down to the monolayer, the absence of interlayer interactions makes electrons in graphene behave as massless Dirac fermions,^[^ ^14^, ^15^, ^16^ ^]^ in sharp contrast to the massive fermions in few layered graphene.^[^ ^8^, ^12^ ^]^ Significant effect of interlayer interactions was also found in other vdW layered materials such as, transition metal dichalcogenides (TMDCs),^[^ ^5^, ^17^, ^18^, ^19^ ^]^ transition metal halides,^[^ ^20^, ^21^, ^22^ ^]^ metal phosphorus trichalcogenides, etc.^[^ ^21^, ^22^, ^23^ ^]^

**Figure 1:**
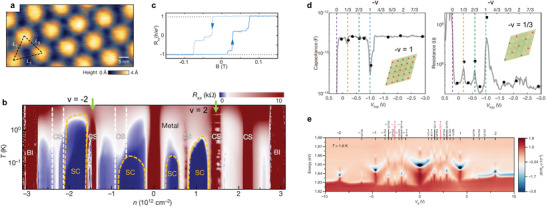
Strongly correlated phenomena induced by interlayer coupling in 2D moiré superlattices. a) Twisted bilayer graphene (TBG) near the magic angle (i.e., MATBG) has a triangular lattice with alternating AA and AB/BA stacking orders in the moiré supercell. AA sites appear higher in the STM topology and hold large local density of states (DOS). b) Phase diagram of MATBG. Correlated states (CS) appear at all the integer fillings of the moiré band, which are accompanied by superconducting (SC) phases. Two green arrows indicate the integer fillings at *v* = ±2. c) Quantum anomalous Hall (QAH) showing quantized Hall resistance in MATBG aligned with hBN at an integer filling *v* = 3. The observed quantized Hall signal at *B* = 0 comes from a pure orbital magnetism and indicates an incipient topological Chern insulator as a result of interaction‐induced spontaneous time‐reversal symmetry breaking. d) Mott insulating states (half‐filling of the moiré hole band at *v* = −1) and generalized Wigner crystal insulating states (fractional fillings at *v* = −1/3 and *v* = −2/3) were observed in angle‐aligned TMDC (WS_2_/WSe_2_) moiré superlattices by an effective capacitance measurement. Insets show the localized hole positions (red balls) on the moiré superlattice at *v* = −1 and *v* = −1/3. e) A diverse set of Wigner crystal states beyond 1/3 and 2/3 fillings also exist in the WS_2_/WSe_2_ moiré system. a) Reproduced with permission.^[^ ^42^ ^]^ Copyright 2019, Springer Nature Limited. b) Reproduced with permission.^[^ ^41^ ^]^ Copyright 2019, Springer Nature Limited. c) Reproduced with permission.^[^ ^61^ ^]^ Copyright 2020, American Association for the Advancement of Science. d) Reproduced with permission.^[^ ^49^ ^]^ Copyright 2020, Springer Nature Limited. e) Reproduced with permission.^[^ ^51^ ^]^ Copyright 2020, Springer Nature Limited.

The successful isolation of 2D layered materials together with the development of vdW assembly technique^[^ ^24^, ^25^ ^]^ have enabled the broad exploration of novel physical phenomena in 2D vdW heterostructures. The formed vdW heterostructures, following the creation of graphene/hexagonal boron nitride (hBN) heterostructures, usually do not have to satisfy the stringent lattice matching requirements, and that the interfaces between the layers can be extremely clean. Since 2018, fabricating artificial moiré superlattices has provided a powerful tool to engineer the electronic band structures and properties of 2D materials that are otherwise difficult by conventional techniques in bulk materials.^[^ ^26^, ^27^, ^28^, ^29^, ^30^, ^31^, ^32^, ^33^, ^34^, ^35^, ^36^, ^37^, ^38^, ^39^ ^]^ Many fascinating new phases of matter have been discovered in 2D moiré superlattices,^[^ ^32^, ^33^, ^40^, ^41^, ^42^, ^43^, ^44^, ^45^, ^46^, ^47^, ^48^, ^49^, ^50^, ^51^, ^52^, ^53^, ^54^, ^55^, ^56^, ^57^, ^58^, ^59^, ^60^, ^61^, ^62^, ^63^, ^64^ ^]^ including Mott and superconductivity ground states in magic‐angle twisted bilayer graphene (MATBG)^[^ ^32^, ^33^, ^40^, ^41^ ^]^ and ABC trilayer graphene aligned with hBN,^[^ ^43^, ^44^ ^]^ and moiré excitons^[^ ^45^, ^46^, ^47^, ^48^ ^]^ and Wigner crystal states^[^ ^49^, ^50^, ^51^, ^52^ ^]^ in TMDC moiré superlattices, which are achieved by a delicate control of doping and/or bandwidth in a single bench‐top device. Interlayer electronic coupling in moiré superlattices leads to the formation of moiré flat minibands.^[^ ^29^, ^53^, ^54^, ^55^, ^56^, ^57^, ^58^, ^59^ ^]^ Consequently, the on‐site and/or long‐range coulomb interactions can become dominant over the kinetic energy and lead to strongly correlated electronic states. 2D vdW moiré superlattices also provide a unique playground for studying the non‐trivial topological properties in a strongly correlated electronic system in which many‐body electron‐electron Coulomb interactions can induce symmetry‐breakings.^[^ ^60^, ^61^, ^62^, ^63^, ^64^ ^]^ Realization of an electrical control of robust magnetic states (pure orbital magnetism)^[^ ^60^, ^61^, ^62^ ^]^ and superconducting switches in working devices^[^ ^65^, ^66^, ^67^ ^]^ delivers promise of strongly correlated 2D moiré superlattices in the application of electrically‐writable magnetic memory and quantum information computing using tunable superconducting circuits. The works displayed in **Figure** **1** represent a summary of recent experimental findings of moiré pattern‐induced correlated physical phenomena in systems of MATBG (Figure 1a–c) and TMDC (Figure 1d,e) vdW heterostructures.

Compared with the exciting developments in 2D vdW moiré superlattices, 1D moiré superlattices have been little explored. In the following, we first introduce 1D moiré superlattices and the interlayer interactions using a representative vdW material in 1D, double‐walled carbon nanotubes (DWNTs). Then we briefly review previous results on the mechanical and transport properties of structure‐identified DWNTs. We then focus on the optical spectroscopy studies of interlayer interactions in structure‐identified DWNTs, which revealed new insights of the interlayer interactions in 1D moiré systems, with highlighting the unique physics of 1D moiré systems. Finally, we will discuss the future directions and outlook of studying the 1D moiré superlattices and 1D moiré physics.

## 2 Interlayer Interactions in 1D Moiré Superlattices

When two or more vdW layered materials are concentrically rolled up, they form 1D moiré superlattices. Double‐walled and multi‐walled carbon,^[^ ^68^, ^69^, ^70^, ^71^, ^72^ ^]^ boron nitride,^[^ ^73^, ^74^ ^]^ TMDC^[^ ^75^ ^]^ nanotubes and their heterostructures^[^ ^76^, ^77^ ^]^ are among the available material list of 1D vdW moiré superlattices.

### 2.1 A Model System of 1D Moiré Superlattices: Double‐Walled Carbon Nanotubes

**Figure 2:**
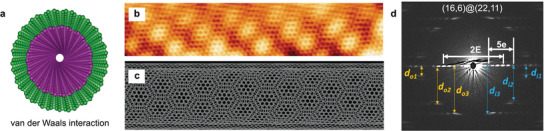
VdW‐coupled 1D DWNT moiré superlattice. a) Structure model of a DWNT composed of two vdW‐coupled SWNTs. b) STM topology image of an isolated DWNT showing a spatially‐modulated moiré pattern. c) The rigid lattice model of a DWNT reproducing the observed moiré pattern in b). SWNTs (25,20) and (21,32) are used in panel c) for the inner and outer nanotubes. d) Electron diffraction pattern of an DWNT with chirality (16,6)@(22,11). There are total 4 sets of hexagons in its diffraction pattern, two each belonging to each nanotube. The equatorial line oscillates in intensity with E and e representing the long and short periods. Distances for features outside the equatorial line to the equatorial line (e.g., *d*_o2_ and *d*_o3_ for the outer SWNT and *d*_i2_ and *d*_i3_ for the inner SWNT) are used to uniquely determine the DWNT chirality in conjunction with the measured E and e. b,c) Reproduced with permission.^[^ ^85^ ^]^ Copyright 2013, ELSEVIER. d) Reproduced with permission.^[^ ^86^ ^]^ Copyright 2014, Springer.

DWNTs provide one of the ideal model systems to study the interlayer interactions in 1D moiré superlattices because the physical properties of a DWNT depend on the combination of the two components,^[^ ^78^, ^79^, ^80^, ^81^ ^]^ non‐interacting single‐walled carbon nanotubes (SWNTs). The electronic property of each SWNT is physically determined by a chiral vector with two integer components (also known as chirality). Conventionally, the two chiral vectors for inner and outer SWNTs in a DWNT are represented by ***C*** and ***C*** ′,^[^ ^78^ ^]^ respectively, where

(1) $$\begin{array}{ccc} & & C=n_{1}a_{1}+n_{2}\;a_{2}=\left(n_{1},n_{2}\right)\\ & & \text{and}\\ & & C^{\prime }\;=n_{1}^{\prime }\;a_{1}+n_{2}^{\prime }\;a_{2}=\left(n_{1}^{\prime },n_{2}^{\prime }\right) \end{array}$$

with ***a***_1_ and ***a***_2_ denoting the unit lattice vectors of 2D graphene sheet. The chiral angle of a specific SWNT is defined as

(2) $$\theta \;=\cos^{-1}\frac{2n_{1}+n_{2}}{2\sqrt{{n_{1}}^{2}+{n_{2}}^{2}+n_{1}n_{2}}}\;$$

constrained within 0° ≤ *θ* ≤ 30°. The physical structure of a DWNT is denoted as ***C*** @ ***C*** ′. In a DWNT, the inner and outer SWNTs are coupled by vdW interactions (**Figure** **2**a) with interlayer distance ranging from 0.30–0.40 nm.^[^ ^82^, ^83^ ^]^ It is still debatable if there is any correlation of chiral angles between the inner and outer SWNTs,^[^ ^82^, ^83^, ^84^ ^]^ and the answer may depend on the synthesis method by which the DWNT samples are produced. The DWNT forms a generic 1D moiré superlattice in analogy with the 2D counterpart. Formation of 1D moiré superlattices has been clearly observed in real space by scanning tunneling microscopy (STM) (Figure 2b,c).^[^ ^85^ ^]^

The physical structure (i.e., moiré superlattice) of an individual DWNT so far can only be unequivocally determined by electron beam diffractions.^[^ ^82^, ^83^, ^84^, ^86^, ^87^, ^88^, ^89^ ^]^Figure 2d shows a typical diffraction pattern of an isolated DWNT and illustrates how the chirality of two constituent SWNTs are determined.^[^ ^86^ ^]^ The diffraction pattern of a DWNT typically exhibits two sets of mutually twisted hexagonal patterns belonging to two constituent SWNTs.^[^ ^90^, ^91^, ^92^ ^]^ The equatorial line in the DWNT diffraction pattern (perpendicular to the DWNT axis in real space) exhibits a “beating‐like” behavior with the fast oscillations (with period e) modulated by the slow ones (with period E). This originates from the interference of electron waves scattered by two nanotubes in the radial direction. Because of the high sensitivity of the wave interference to diameter and/or chirality of each nanotube, the equatorial line profile serves as a decisive feature for the unambiguous determination of the DWNT structure. By measuring the distances from features outside the equatorial line to the equatorial line, for example, *d*_o2_ and *d*_o3_ from outer SWNT and *d*_i2_ and *d*_i3_ from inner SWNT, the DWNT physical structure can be fully obtained by using a set of equations:^[^ ^89^ ^]^

(3) $$\begin{array}{ccc} & & n_{1}=\frac{\pi }{\sqrt{3}}\left(2d_{i3}-d_{i2}\right)\left(\frac{1}{e}-\frac{1}{E}\right),n_{2}=\frac{\pi }{\sqrt{3}}\left(2d_{i2}-d_{i3}\right)\left(\frac{1}{e}-\frac{1}{E}\right),\\ & & n_{1}^{{}^{\prime }}=\frac{\pi }{\sqrt{3}}\left(2d_{o3}-d_{o2}\right)\left(\frac{1}{e}+\frac{1}{E}\right),n_{2}^{{}^{\prime }}=\frac{\pi }{\sqrt{3}}\left(2d_{o2}-d_{o3}\right)\left(\frac{1}{e}+\frac{1}{E}\right) \end{array}$$

The determined structure of Figure 2d using Equation (3) is (16,6)@(22,11),^[^ ^86^ ^]^ whose result is further compared and corroborated by simulations.

Earlier theoretical studies have focused on the commensurate DWNTs or SWNT bundles comprising armchair or zigzag SWNTs, showing significant changes of the band structure with interlayer interactions switched on.^[^ ^93^, ^94^, ^95^, ^96^ ^]^ However, such commensurate DWNTs have been very rarely observed experimentally.^[^ ^84^ ^]^ For the commonly observed incommensurate DWNTs conventional wisdom suggests that the interlayer interaction is very weak. This is because the coupling matrix element is averaged out to zero when summing up the oscillating interlayer hopping at each lattice site.^[^ ^97^, ^98^ ^]^

1D materials such as, SWNTs show characteristic paired subbands associated with van Hove singularity (VHS) of density of states (DOS).^[^ ^78^ ^]^ This unique property makes the optical spectroscopy tool very powerful in probing the interlayer interactions in 1D systems. In fact, early photoluminescence studies consistently revealed energy redshifts of optical transition energies for the inner semiconducting SWNTs in DWNTs^[^ ^99^, ^100^, ^101^, ^102^ ^]^ and SWNTs embedded in a dielectric environment.^[^ ^103^, ^104^ ^]^ The environmental screening effect is accounted for the observed energy redshifts.^[^ ^105^, ^106^, ^107^ ^]^ These results support the weak coupling in incommensurate DWNTs where one SWNT wall behaves no more than a dielectric screening layer of the other SWNT.

Before focusing on the new optical spectroscopy discoveries in interlayer electronic interactions in DWNTs, we first briefly review the works on the interlayer mechanical coupling as well as on the transport studies in the structure‐identified DWNTs, showing signs of non‐trivial interlayer coupling.

### 2.2 Mechanical Coupling in Double‐Walled Carbon Nanotubes

**Figure 3:**
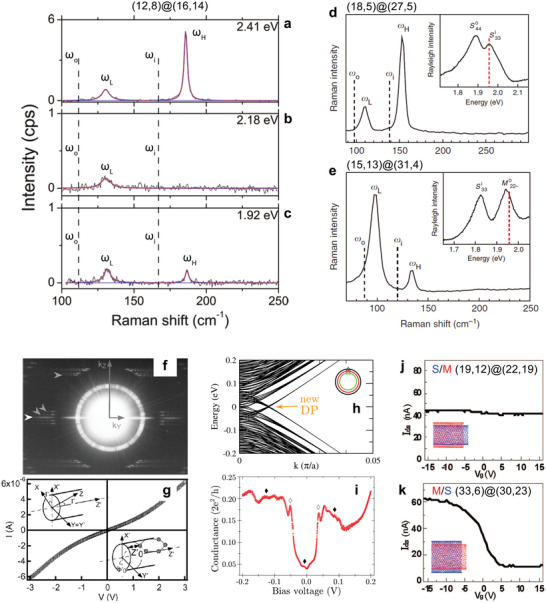
Mechanical and electrical transport properties of structure‐identified DWNTs. a–c) Raman RBMs for a (12,8)@(16,14) DWNT with laser excitations at 2.41, 2.18, and 1.92 eV, respectively. Excitations at 2.41, 2.18, and 1.92 eV are in close vicinity to S_33_ of inner (12,8), S_44_ of outer (16,14), and S_33_ of outer (16,14) nanotubes, respectively. The observed coupled modes *ω*_L_ and *ω*_H_ are very different from those of uncoupled SWNTs (*ω*_i_ and *ω*_o_ indicated by the dashed lines). d,e) Raman RBMs for (18,5)@(27,5) and (15,13)@(31,4) DWNTs, respectively, with a single laser excitation at 1.96 eV (red dashed line in the inset). f) Electron beam diffraction pattern of a semiconducting DWNT (51,34)@(53,43). g) *I*–*V* curve of the same DWNT in f) measured inside a transmission electron microscope (TEM) in ultrahigh vacuum. Linear *I*–*V* near zero bias indicates a finite DOS near the Fermi surface. h) Tight‐binding band structure of a zigzag TWNT (1045, 0)@(1054, 0)@(1063, 0), where the formation of a new Dirac point (DP) is indicated by an arrow. i) Measured tunneling conductance as a function of bias voltage between an isolated TWNT and metal contact at 4.2 K. The observed conductance singularities (white diamonds) and oscillations (black diamonds) are assigned to the opening of new DPs caused by the formed 1D moiré superlattice. j,k) Gate‐dependent current of two structure‐identified DWNTs (19,12)@(22,19) and (33,6)@(30,23). The effect of interlayer electronic interactions is not noticeable. a–c) Reproduced with permission.^[^ ^112^ ^]^ Copyright 2011, American Chemical Society. d,e) Reproduced with permission.^[^ ^113^ ^]^ Copyright 2013, Springer Nature Limited. f,g) Reproduced with permission.^[^ ^117^ ^]^ Copyright 2002, American Physical Society. h,i) Reproduced with permission.^[^ ^118^ ^]^ Copyright 2016, Springer Nature Limited. j,k) Reproduced with permission.^[^ ^119^ ^]^ Copyright 2009, American Chemical Society.

Raman spectroscopy serves as a valuable tool in studying the vibrational properties and characterizing graphitic materials.^[^ ^108^, ^109^ ^]^ It is widely used to characterize the diameters and chirality information of SWNTs,^[^ ^78^ ^]^ DWNTs,^[^ ^110^ ^]^ and multi‐walled carbon nanotubes (MWNTs)^[^ ^111^ ^]^ in which the observed radial breathing modes (RBMs) can be directly connected with the diameter of each nanotube by *ω*_RBM_ = 228/*d* , where *ω*_RBM_ is in cm^–1^ and *d* is in nm.^[^ ^78^ ^]^ However, the use of such empirical relation was later found problematic when carefully examining the individual DWNTs with known chirality.^[^ ^112^, ^113^ ^]^ **Figure** **3**a–c investigated the Raman RBMs on a (12,8)@(16,14) DWNT with varying laser excitation energies.^[^ ^112^ ^]^ The results shown in Figure 3d,e examined different chirality‐identified DWNTs with a single laser line excitation.^[^ ^113^ ^]^ Both reported that the RBM positions in DWNTs (denoted by *ω*_L_ and *ω*_H_) are very different from those expected from isolated inner and outer SWNTs of the same chirality (*ω*_i_ and *ω*_o_ indicated by the black dashed lines). These two studies also found that *ω*_L_ and *ω*_H_ appeared whenever the excitation hits on the optical resonance in either of two nanotubes (Figure 3b, however, does not hold). The coupled modes (i.e., in phase and out of phase modes) can be theoretically described by two coupled mechanical oscillators.^[^ ^113^, ^114^ ^]^ These observations show clear evidences of a strong mechanical coupling between the vdW‐coupled SWNTs, making people aware of DWNT distinct vibrational properties than simply adding up those of two component SWNTs. Similar effect of the mechanical coupling was also reported for the G modes at higher wavenumbers.^[^ ^115^ ^]^ Evidence of moiré‐induced vibrational coupling in DWNTs was recently reported,^[^ ^116^ ^]^ highlighting the importance of 1D moiré superlattices.

### 2.3 Electrical Transport Studies in Double‐Walled Carbon Nanotubes

Transport measurement is suitable to probe the electronic band structure at the Fermi energy. The first experimental evidence showing the possible change of DWNT band structure was reported in 2002 by an in‐situ transport study (Figure 3f,g).^[^ ^117^ ^]^ The authors observed very puzzling finite DOS at the Fermi surface (zero bias region in the *I*–*V* curve shown in Figure 3g), which normally should be gapped for a DWNT (51,34)@(53,43) consisting of both semiconducting inner and outer SWNTs (Figure 3f). It indicated the possibility of notable band structure change induced by the interlayer electronic interactions, although the exact mechanism was not understood. A more recent transport (tunneling) study (Figure 2h,i) reported extra conductance singularities and oscillations in an isolated MWNT at 4.2 K (indicated by white and black diamonds in Figure 2i).^[^ ^118^ ^]^ Band structure shown in Figure 2h was calculated using a hypothesized MWNT structure. The authors argued the moiré superlattice‐induced opening of new Dirac points (DP) (Figure 2h) accounts for the appearance of these additional conductance in Figure 2i. This is a very interesting result, but the lack of definitive structure information and the complicated contact between the MWNT and metal leads make a definitive physical interpretation difficult. There were other transport studies on structure‐identified individual DWNTs prepared by either a direct growth^[^ ^119^ ^]^ or sorted technique.^[^ ^120^, ^121^ ^]^ Typical results of gate‐dependent conductance are shown in Figure 3j,k.^[^ ^119^ ^]^ The observed transfer characteristics can be mostly described by outer shell‐contacted DWNTs in S@M (Figure 3j) and M@S configurations (Figure 3k) without considering interlayer electronic coupling.^[^ ^119^, ^120^, ^121^, ^122^ ^]^ In summary, transport measurements reported so far have not found conclusive evidence of the effect of 1D moiré superlattices in DWNTs.

## 3 Optical Spectroscopy Studies on Interlayer Interactions in Double‐Walled Carbon Nanotubes

### 3.1 Interactions in the Perturbative Regime

**Figure 4:**
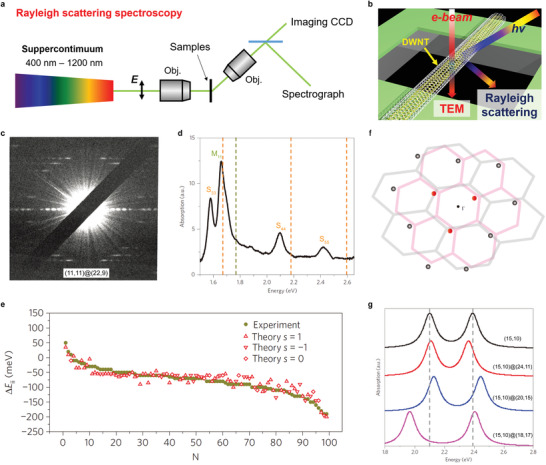
Interlayer electronic interactions probed by optical spectroscopy for structure‐identified incommensurate DWNTs. a) Schematic of Rayleigh scattering spectroscopy setup used to probe the optical transitions of nanotubes. b) Experimental scheme showing the combined optical spectroscopy and electron beam diffraction on individual DWNTs. c) Electron beam diffraction pattern of an DWNT with chirality (11,11)@(22,9). d) Absorption spectrum of the same DWNT shown in panel c). Three optical transitions (S_33_, S_44_, and S_55_) come from the outer semiconducting SWNT (22,9) and one (M_11_) comes from the inner metallic SWNT (11,11). Comparing to the optical transitions of isolated SWNTs (22,9) and (11,11) (indicated by the dashed lines), all the four optical transitions in DWNT exhibit energy redshifts. e) Summary of energy shifts for 99 optical transitions in different DWNTs. Experimental results are presented by solid circles, and the predicted values using the developed perturbation theory are shown by red markers. f) Schematic drawing of extended 2D GBZ depicting the physics of interlayer electronic interactions between the inner (pink) and outer (black) nanotubes. Here the GBZ of outer layer (black) is uniaxially distorted to satisfy the electronic coupling conditions (see the main text). Important electronic states included in the perturbation theory (Equation (4)) are indicated by the red circles which are the three closest states around Γ point; contribution from other possible states is small and indicated by the black circles. g) Simulation of the interlayer electronic interaction‐induced optical transitions (S_33_ and S_44_) for a (15,10) inner SWNT with varying the chirality of outer SWNT using Equation (4). b) Reproduced with permission.^[^ ^86^ ^]^ Copyright 2014, Springer Nature. c‐g) Reproduced with permission.^[^ ^98^ ^]^ Copyright 2014, Springer Nature Limited.

Progress continued after successful applications of broadband optical spectroscopy technique (either Rayleigh scattering spectroscopy or absorption spectroscopy) on individual SWNTs and DWNTs suspended in air.^[^ ^123^, ^124^, ^125^, ^126^, ^127^, ^128^, ^129^, ^130^ ^]^ Typical samples are directly grown by chemical vapor deposition method across a long trench on SiO_2_/Si substrates with a careful density control.^[^ ^131^, ^132^ ^]^ **Figure** **4**a shows the schematic of Rayleigh scattering spectroscopy technique used to probe the optical resonances of nanotubes. Broadband light from a supercontinuum laser source (e.g., 1.2–2.7 eV) is focused on the suspended nanotube samples with laser polarized along the nanotube axis. The light scattered by the nanotube is collected by a second objective and directed to a CCD camera for imaging and a spectrograph for spectroscopy measurement. The optical transition energies coming from the paired 1D subbands (denoted by S_11_, S_22_, M_11_, S_33_, etc.) in each SWNT can be precisely determined by using the optical spectroscopy technique. The combined broadband spectroscopy and electron microscopy (determine the chirality) on the individual nanotube level (schematic drawing shown in Figure 4b) enable a clear observation of the effect of interlayer electronic coupling when comparing to the established optical spectra of the corresponding SWNTs of the same chirality.^[^ ^81^ ^]^

Figure 4c–g show the results from ref. [98] of the optical resonances in chirality‐defined DWNTs. The experimentally measured optical absorption of an DWNT is shown in Figure 4d whose structure has been identified as (11,11)@(22,9) (diffraction pattern shown in Figure 4c). Four absorption peaks in Figure 4d are identified: three peaks come from the optical transitions (S_33_, S_44_, S_55_) of the outer semiconducting nanotube with chirality (22,9) and the other peak (M_11_) comes from the inner metallic nanotube with chirality (11,11). All the peaks exhibit energy redshifts when comparing with the corresponding SWNTs of the same chirality (dashed lines in Figure 4d). The statistical result of energy shifts for 99 optical transitions is presented in Figure 4e (solid circles). It is clear that both energy red and blue shifts (−200 meV to +50 meV) exist, being consistent with other reports.^[^ ^86^ ^]^

**Figure 5:**
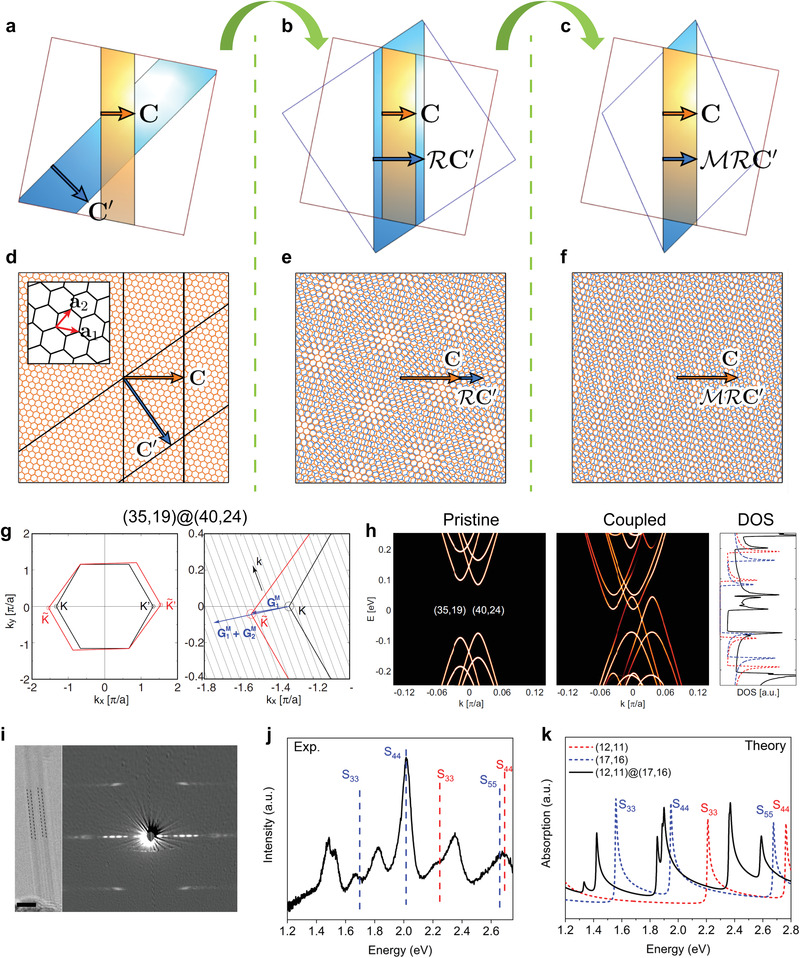
Theoretical model and experimental observation of non‐perturbative electronic interactions in 1D DWNT moiré superlattices. a,d) Schematic drawing and lattice structure of two AA‐stacked graphene nanoribbons. b,e) Schematic drawing and 2D moiré superlattice structure after a rotation operation of the outer ribbon (*R**C'***) such that ***C*** and ***C'*** align. c,f) Schematic drawing and 1D moiré superlattice structure after a consecutive uniaxial compression of the outer ribbon (*MR* ***C'***). The inner ribbon is presented in orange (orange lattice) with ***C*** denoting the chiral vector of the inner SWNT, while the outer ribbon is shown in blue (blue lattice) with ***C'*** denoting the chiral vector of the outer SWNT. g) 2D GBZ and its enlarged view of a DWNT (35,19)@(40,24) that satisfies the strong‐coupling condition exactly (i.e.,$\Delta K=G_{1}^{M}$). $G_{i}^{M}$ is the reciprocal lattice vector of DWNT moiré superlattices defined in Equation (5). h) Calculated band structures of two pristine SWNTs (35,19) and (40,24) (left panel) and the coupled DWNT (35,19)@(40,24) (middle panel). The corresponding DOS plot is shown on the right panel with dashed lines denoting the DOS of two pristine SWNTs and solid black line denoting the DOS of the coupled DWNT. i) TEM image and diffraction pattern of a DWNT satisfying the predicted strong‐coupling condition. The structure of the DWNT is determined as (12,11)@(17,16) by analyzing the diffraction pattern and further corroborated with simulations. The scale bar in the TEM image is about 2 nm. j) Measured Rayleigh scattering spectrum of the DWNT (12,11)@(17,16). Optical transitions from two pristine SWNTs (17,16) and (12,11) are indicated by the blue and red dashed lines, respectively. The optical spectrum observed is very different from the sum of two constituent SWNTs, indicative of moiré superlattice‐induced strong electronic coupling. k) Theoretically calculated optical absorption spectrum of (12,11)@(17,16) with (solid black line) and without (blue and red dashed lines) the moiré effect within the proposed theoretical framework.^[^ ^133^ ^]^ Note that the calculated optical absorption peaks of pristine SWNTs very well reproduced the experiments within an accuracy of ≈100 meV (Figure 5j,k). a–h) Reproduced with permission.^[^ ^133^ ^]^ Copyright 2015, American Physical Society. i–k) Reproduced with permission.^[^ ^134^ ^]^ Copyright 2020, American Physical Society.

The observed energy blue shift is not compatible with the dielectric screening effect used to explain the previously reported energy redshifts.^[^ ^99^, ^100^, ^101^, ^102^, ^103^, ^104^, ^105^, ^106^, ^107^ ^]^ The authors further proposed the first theoretical model to explain and simulate the observed energy shifts in 1D incommensurate DWNTs.^[^ ^98^ ^]^ The model is based on a calculation of electronic coupling matrix element *M*_*αβ*_ = 〈Ψ_*α*_|*H*_INT_|Ψ_*β*_〉  between the tight‐binding electronic wavefunctions of inner (Ψ_*α*_) and outer (Ψ_*β*_) nanotubes. Shown in Figure 4f, the extended 2D graphene Brillouin zone (GBZ) of outer layer (black) is rotated and uniaxially stretched (compressed in real space) along the nanotube circumferential direction (***C*** and ***C*** **′** directions in **Figure** **5**b) relative to the unchanged GBZ of the inner layer (pink). In this case, the electronic states (in k space) from inner (q_*α*_) and outer SWNT (q_*β*_) can have electronic coupling, that is, the states on the cutting lines from two nanotubes match exactly (see the right panel in Figure 5g). With the knowledge of *M*_*αβ*_ and by using the perturbation theory (to the second order), the energy shifts of, for example, the inner optical transitions Δ*E*_ii_ caused by the outer layer can be calculated as:

(4) $$\Delta \;E_{\mathrm{ii}}=\sum_{\beta =1}^{3}\frac{{\left|M_{\alpha \beta }\right|}^{2}}{E_{\alpha }-E_{\beta }}\;$$

where *E*_*α*_ and *E*_*β*_ are eigen energies of electronic states q_*α*_ (inner) and q_*β*_ (outer) with q_*β*_ = q_*α*_  + *G*_i_ and *G*_i_ the reciprocal lattice vector of pristine graphene. Note that the sum in Equation (4) is taken over three states of the outer nanotube that is closest to Γ point (three red circles in Figure 4f) as an approximation because of the small contribution from other states (black circles in Figure 4f) abiding to the exponential decay of the coupling strength. This theory can reproduce the experimental results reasonably well as shown in Figure 4e. As an example, Figure 4g presents the simulation of the optical transitions (S_33_ and S_44_) for a (15,10) inner SWNT with varying the chirality of outer SWNT. It shows that the amplitude of the energy shift depends sensitively on the specific optical transitions and the outer tube species, which can be either positive or negative with a magnitude as large as 150 meV.

### 3.2 Interactions in the Non‐Perturbative Regime

The interlayer interactions in DWNT moiré superlattices discussed above are still limited within a perturbative regime. On the other hand, a recent work,^[^ ^133^ ^]^ motivated by ref. [135], theorized and calculated the band structure of certain species of DWNTs with moiré superlattice effect beyond the perturbative coupling regime. The schematics and lattice structures presented in Figure 5a–f illustrate how a 1D DWNT moiré superlattice (Figure 5c,f) is constructed from two AA‐stacked graphene nanoribbons (Figure 5a,d). First, align two chiral vectors ***C*** and ***C*** ′ by rotating the outer ribbon relative to the inner one (Figure 5b,e) in which the rotation angle is determined by the chiral angle difference between two SWNTs (Equation (2)). It is followed by a uniaxial compression of the outer ribbon such that ***C*** ′ becomes equal to ***C*** in length (Figure 5c,f). The formed 1D moiré superlattice (Figure 5f) is distinct from the 2D moiré pattern of twisted bilayer graphene (TBG) (Figure 5e) due to the unique uniaxial compression (or stretching) in 1D case.^[^ ^98^, ^133^, ^136^, ^137^, ^138^ ^]^ The chiral vectors of the inner and outer tubes have different lengths, but their registry along the circumferential direction should be commensurate. This makes the lattice structure “virtually” strained along that direction (Figure 5f) and enables some interactions which are not allowed in the stack of two hexagonal lattices in 2D (e.g., the strong‐coupling case in 1D DWNTs to be discussed later). Note that the uniaxial deformation is not an actual lattice deformation; it is the deformation coming from the relative coordinate matching due to the difference between the radii of the inner and outer nanotubes. In k space, it results in a rotated and uniaxial stretched GBZ for the outer layer with an intact GBZ for the inner layer (also see Figure 4f). We also note that the intertube distance of DWNTs can vary over a wider range between 0.30 and 0.40 nm^[^ ^82^, ^83^ ^]^ depending on the combination of the chiralities, while the interlayer distance of TBG only slightly varies with the twist angle.^[^ ^139^ ^]^ The DWNTs with shorter intertube distance will have much stronger interactions than those with longer distance.

Within the theoretical framework proposed in ref. [133], the interlayer electronic coupling in a DWNT moiré superlattice is fully characterized by the relative orientation between ***C*** and ***C*** ′. The reciprocal lattice vectors in DWNT moiré superlattice have the following form

(5) $$G_{i}^{M}\cdot C\;=\;2\pi \left(n_{i}-n_{i}^{\prime }\right)\left(i\;=\;1,2\right)$$

where ***C***, $n_{i}$ , and $n_{i}^{\prime }$ are defined in Equation (1). In Figure 5g the 2D GBZ of the outer layer is presented by the distorted red hexagon (Dirac points $\tilde{K}$ and ${\tilde{K}}^{\prime }$) while that of the inner layer is shown by the undistorted black hexagon (Dirac points *K* and *K*′). The states of the nanotubes are quantized, resulting in the cutting lines shown in Figure 5g. Significant modification of the band structure can take place under two different conditions, which are dubbed the strong‐coupling case and the flat‐band case, respectively. The former occurs when ***C*** and ***C*** ′ are nearly parallel to each other and, at the same time, the difference of two chiral vectors (***C*** ′ – ***C***) is parallel to the armchair direction. Then the moiré superlattice potential makes the resonant coupling between the states of constituent nanotubes near Dirac points, leading to a drastic energy shift and splitting of the subband edges. The latter case occurs under the condition that ***C*** and ***C*** ′ are nearly parallel, and ***C*** ′ − ***C*** is parallel to the zigzag direction. A long‐period moiré interference potential turns the original single nanotube bands into a series of nearly flat bands, reminiscent of the moiré flat bands in TBG at a small twist angle.^[^ ^29^, ^133^ ^]^ The presence of the strong‐coupling and flat‐band cases is a direct consequence of the strained structure along the circumferential direction that is unique to 1D superlattices.

We present below a simple derivation of the strong‐coupling case. The interacting Hamiltonian for the low‐energy electrons can be written as

(6) $$H\;=\left(\begin{array}{cc} H_{1}\left(k\right) & U^{†}\\ U & H_{2}\left(k\right) \end{array}\right)\;$$

where the situation around one valley (e.g., *K* valley) is considered. *U* is the interlayer coupling matrix element with the form $\left\langle k^{\prime },X_{l^{\prime }}^{\prime }|T|k,X_{l}\right\rangle$, where | ***k***, *X_l_* 〉 is an intralayer Bloch wave basis, *X* and *X*′ are either A or B atom in graphene, *l* and *l*′ are either 1 or 2 (inner or outer layer), and *T* is the interlayer coupling Hamiltonian. If the formed moiré period is much longer than the graphene lattice (continuum model), the interlayer electronic coupling *U* can be simplified, consisting of three Fourier components of 1, $e^{iG_{1}^{M}\cdot r}$ and $e^{i(G_{1}^{M}+G_{2}^{M})\cdot r}$. The coupling effect will be significant when the distance between *K* and $\tilde{K}$ points,

(7) $$\Delta K\equiv \tilde{K}-K\;=\;\left(2G_{1}^{M}+G_{2}^{M}\right)/3$$

is close to any of the three Fourier components of 0, $G_{1}^{M}$and $G_{1}^{M}+G_{2}^{M}$. This mechanism leads to the criteria showing the strong‐coupling effect, which was recently observed in an experiment (discuss later). A semiconducting DWNT with chirality (35,19)@(40,24) matches the predicted condition of strong‐coupling, that is, $\Delta K\;=G_{1}^{M}\;$ (Figure 5g). Its band structure (Figure 5h) shows a drastic change: the original gap vanishes, exhibiting a finite DOS at the Fermi energy and extra number of VHS in the DOS plot shown on the right. We note here that, in Equation (4), one explicitly uses just one band and implementes the effect of the other three bands (three dominant states in the other layer) perturbatively. Their effect is simplified by the constants *M*_*αβ*_ in that procedure, and the higher order terms are all neglected. On the other hand, in Equation (6), the strong‐coupling effect predicted by the non‐perturbative approach explicitly uses two bands. Thus, at least the interaction between these two bands are precisely described and no higher order terms are neglected.

A very recent experiment reported the drastic change of electronic structure in a DWNT moiré superlattice.^[^ ^134^ ^]^ The experiment was carried out by a combined Rayleigh scattering spectroscopy and electron beam diffraction on individual ultraclean DWNTs suspended in air (experimental scheme shown in Figure 4b). The Rayleigh scattering spectra are obtained by normalizing the measured scattered light from nanotubes to the incident laser profile. Figure 5i shows the transmission electron microscopy (TEM) image and the diffraction pattern of this special DWNT. The chiral index is unequivocally identified as (12,11)@(17,16) from the diffraction pattern, which is further corroborated by the systematic simulations. The determined structure matches the theoretical criteria of the strong‐coupling exactly since ***C*** and ***C*** ′ are both nearly armchair with a chiral angle difference of about 0.4° and that ***C*** ′ − ***C *** = (5, 5)  orients along the armchair direction. Eight well‐defined optical resonances are observed within the energy 1.35‐2.7 eV in its optical transition spectrum (Figure 5j). The expected optical transition energies of two pristine SWNTs comprising the DWNT are indicated by the dashed blue (outer SWNT) and red (inner SWNT) lines.^[^ ^81^ ^]^ At a glimpse, the optical spectrum is very puzzling because the peaks are completely different from the optical transitions of two constituent SWNTs. Several additional optical resonances emerge in the DWNT optical spectrum, which definitely cannot be explained by the perturbation theory (e.g., Equation (4)). Using the theoretical framework including the non‐perturbative moiré superlattice effects,^[^ ^133^ ^]^ the calculated optical absorption spectrum of (12,11)@(17,16) is shown as the solid black line in Figure 5k. The theoretically calculated optical spectrum qualitatively reproduces the experiment (Figure 5j). The calculated absorption spectra of pristine SWNTs (12,11) and (17,16) are also shown by the dashed red and blue lines, respectively. Note that the calculated optical absorption peaks of pristine SWNTs very well reproduce the experiments within an accuracy of ≈100 meV (Figure 5j,k). The theory shows that the moiré superlattice‐induced interlayer coupling strongly mixes the electronic states in two SWNTs, resulting in a very different band structure and thus a drastic change of the optical spectrum.

**Figure 6:**
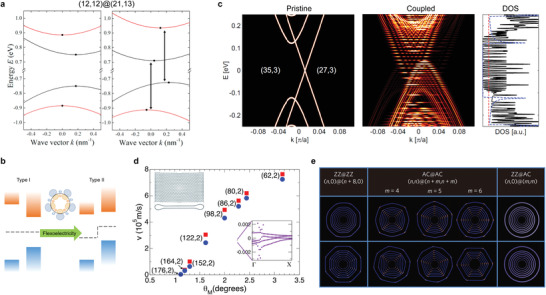
Theoretical prediction and understanding of the physical phenomena related to 1D moiré superlattices. a) Dispersion relation of a DWNT (12,12)@(21,13) without (left panel) and with (right panel) the effect of moiré superlattices. Dispersion curves of inner and outer nanotubes are shown in red and black, respectively. VHS positions are indicated by the black dots in which VHS of pristine outer SWNT is taken as the origin (*k* = 0). The interlayer coupling shifts the VHS positions, which leads to the inter‐tube optical transitions (indicated by the arrows). b) Flexoelectricity‐induced change of band alignment from type I to type II predicted for DWNTs with diameters larger than 2.4 nm. c) Formation of flat bands in vdW‐coupled DWNT with chirality (27,3)@(35,3). d) Calculated Fermi velocities as a function of twist angle (*θ*_M_) between top and bottom layers in FNTs (structure model shown in the upper left inset). The velocity of the collapsed (176,2) SWNT reduces to zero at *θ*_M_ ≈ 1.12°. The inset on the bottom right presents the enlarged view of the band structure of FNT collapsed from a (176,2) SWNT. It shows moiré bands are very flat with a bandwidth smaller than ≈1 meV. e) Circumferential faceting of achiral double‐walled nanotubes (carbon and boron nitride nanotubes). It tends to happen when the chiral angles between inner and outer nanotubes match, that is, both inner and outer nanotubes are armchair (AC) or zigzag (ZZ). This kind of lattice reconstruction can impact the electronic properties of 1D moiré superlattices. a) Reproduced with permission.^[^ ^140^ ^]^ Copyright 2020, American Physical Society. b) Reproduced with permission.^[^ ^143^ ^]^ Copyright 2020, American Chemical Society. c) Reproduced with permission.^[^ ^133^ ^]^ Copyright 2015, American Physical Society. d) Reproduced with permission.^[^ ^144^ ^]^ Copyright 2020, American Chemical Society. e) Reproduced with permission.^[^ ^145^ ^]^ Copyright 2016, Springer Nature.

Several theoretical reports quickly followed the above observation of 1D moiré superlattice effect.^[^ ^140^, ^141^, ^142^, ^143^, ^144^ ^]^ One study predicted strong inter‐tube optical transitions in DWNT moiré superlattices when satisfying certain structure requirements.^[^ ^140^ ^]^ It claimed that the interlayer electronic coupling can modify the band structure in quite a few DWNTs and shift the VHS positions of each constituent SWNTs (**Figure** **6**a), making the inter‐tube transitions optically bright and dominate over the intra‐tube transitions (indicated by the arrows in the right panel of Figure 6a). It is now well accepted that new optical features can emerge in DWNTs due to strong coupling of interlayer electronic states.

## 4 Future Outlook and Summary

There are several important questions to be answered in the study of 1D moiré superlattices.

### 4.1 Moiré Excitons

Does well‐defined moiré excitons exist in DWNTs and other 1D superlattices, and are they spatially localized? The normal band alignment in a DWNT is type I, that is to say, the conduction and valence bands of the outer SWNT energetically lie within those of the inner SWNT. Therefore, in principle, only intralayer excitons (i.e., electron and hole reside in the same SWNT) can exist if we neglect the band structure reconstruction.^[145]^ The intralayer excitons can be preferentially trapped by the moiré superlattice potential at certain regions inside the moiré unit cell where a global or local energy minimum is located.^[^ ^56^, ^146^, ^147^ ^]^ Exciton localization due to the moiré effect has been observed in 2D TMDC moiré superlattices.^[^ ^148^, ^149^ ^]^ The realization of 1D moiré excitons can potentially realize the attractive 1D array of zero‐dimensional (0D) quantum emitters. On the other hand, Type II band alignment is recently predicted to exist for large diameter DWNTs above a threshold diameter of 2.4 nm owing to the flexoelectricity effect between the inner and outer nanotubes (Figure 6b).^[^ ^143^ ^]^ This can potentially facilitate the formation of interlayer excitons and collection of photo‐excited carriers in DWNTs and optoelectronic devices made from them. Heteronanotubes with different chemical compositions such as MoS_2_/WS_2_ can be intriguing systems to investigate moiré excitons in 1D as the study of 2D TMDC moiré superlattices have revealed rich correlated physics.

### 4.2 Moiré Flat Bands

Can we realize 1D moiré flat bands and new correlated quantum phases in 1D moiré superlattices? Flat band formation in DWNT moiré superlattices has been theoretically predicted as shown in Figure 6c due to the periodical moiré potential with a long period^[^ ^133^ ^]^ (a more generalized model was recently presented^[^ ^150^ ^]^). In this example, the chiral vectors for inner (27,3) and outer (35,3) nanotubes are nearly parallel to each other with their difference orientating along the zigzag direction, which matches the criteria to generate the flat‐band case in DWNT moiré superlattices. In such a flat‐band case, the on‐site and long‐range electron‐electron Coulomb interactions can dominate over the kinetic energy such that insulating behavior at half‐ and fractional fillings can occur, forming the Mott and generalized Wigner crystal states in 1D, like the case in 2D. Very interestingly, like the MATBG, similar magic‐angles to have flat moiré bands and vanishing Fermi velocities were recently predicted in another 1D moiré system, flattened carbon nanotubes (FNTs) (Figure 6d).^[^ ^144^ ^]^ This hypothesized structure is transformed from a collapsed large‐diameter nearly‐zigzag SWNT (structure model shown in the upper left inset of Figure 6d). Notably, FNTs that are collapsed from nearly‐zigzag nanotubes can even have narrower bandwidths (≈1 meV) than MATBG does (inset on the bottom right of Figure 6d), which potentially makes it a stronger correlated electronic system. Experimentally, FNT samples can be produced by either unzipping of DWNTs^[^ ^151^, ^152^ ^]^ or extraction of inner shells of MWNTs,^[^ ^153^ ^]^ being the former with disconnected edge structures. 1D superconductivity in vdW materials may be possibly discovered in 1D moiré superlattices when changing the doping within the moiré flat bands. Moreover, electrons confined in 1D generally exhibits the well‐known Luttinger liquid behavior where the motion of electrons are strongly correlated and only collective excitations are in principle allowed.^[^ ^154^, ^155^ ^]^ 1D moiré superlattices may provide a unique system to investigate the interplay of the two problems of electron correlation from both experiments and theoretical perspectives.

Electrical transport will be the most suitable technique to experimentally probe the correlated phenomena in 1D moiré superlattices near the Fermi energy. A critical component in the transport measurement is the ability to fabricate ultraclean samples and electrical devices. However, the hBN capping technique that is widely used in fabricating high‐quality 2D heterostructures cannot be easily transferred to 1D. People have successfully worked out a device configuration in which nanotube samples were directly grown on pre‐patterned electrodes.^[^ ^156^, ^157^ ^]^ Combing the direct growth and vdW assembly technique is an alternative and practically useful route to fabricate clean devices made from 1D structures.^[^ ^158^, ^159^ ^]^ An even bigger challenge is to find the target sample showing flat‐band physics; this difficulty is mainly caused by the inability to controlling the structure of 1D vdW superlattices during the sample growth.

### 4.3 Strain and Structural Reconstruction

What is the influence of strain and lattice reconstruction on the electronic properties of 1D moiré superlattices? The present research of 2D moiré superlattices suffers from the sample inhomogeneity across different domains.^[^ ^160^, ^161^ ^]^ The variation of twisting angles over micrometer scale is believed to come from the non‐uniform stain induced in the vdW stacking process,^[^ ^162^, ^163^ ^]^ which is very difficult to be satisfactorily solved. One intriguing possibility in 1D moiré is that it may allow higher homogeneity of moiré period over micrometer length scale since they are prepared by a direct growth technique. This has been partially supported by electron beam diffraction data of long suspended DWNTs.^[^ ^86^, ^119^ ^]^ On the other hand, lattice relaxation driven by the competing interlayer vdW coupling gain and intralayer strain cost may also happen in 1D moiré superlattices. People have identified the effect of circumferential faceting^[^ ^145^, ^164^, ^165^ ^]^ in carbon (middle row) and boron nitride (bottom row) nanotubes when chiral angles match in different nanotube shells (Figure 6e).^[^ ^145^ ^]^ Lattice reconstruction and its effect on the electronic properties have been recently noticed in 2D moiré systems.^[^ ^166^, ^167^, ^168^, ^169^, ^170^, ^171^, ^172^ ^]^ However, so far, little is known for lattice reconstruction in 1D moiré superlattices. In a recent experiment reporting the moiré superlattice effect on a DWNT,^[^ ^134^ ^]^ the optical absorption spectrum was well reproduced by a theoretical model which includes a lattice reconstruction effect following the method for the 2D TBG.^[^ ^171^, ^172^ ^]^

### 4.4 Characterization Tools

Developing convenient tools to directly characterize 1D moiré superlattices is the key to rapidly expand this research field and to correlate the measured properties with the moiré structures. Scanning probe microscopy with high throughput such as, scanning electron microscopy,^[^ ^173^ ^]^ piezoelectric force microscopy,^[^ ^174^ ^]^ conductive atomic force microscopy,^[^ ^175^ ^]^ and scanning near‐field optical microscopy^[^ ^176^ ^]^ are available choices which have been exploited in characterizing the 2D counterparts. Direct visualization of the moiré flat bands in k space was recently achieved in 2D moiré superlattices with the advent of angle‐resolved photoemission spectroscopy (ARPES) with a nanometer‐sized beam.^[^ ^58^, ^59^ ^]^ Its application to 1D moiré superlattices can be very important.

### 4.5 Expansion of 1D Moiré Systems

**Figure 7:**
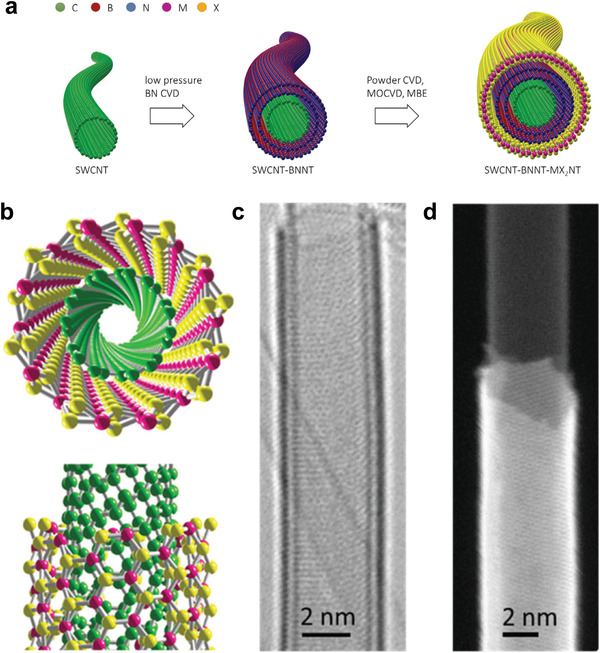
Synthesis of 1D vdW heterostructures. a) Scheme of the proposed growth approach of 1D vdW heterostructures. Nanotubes comprised of different chemical compositions may be realized via the layer‐by‐layer growth. b–d) Structure models, high‐resolution TEM (HRTEM) image and high‐angle annular dark field (HAADF) scanning‐TEM image of a single‐walled MoS_2_ nanotube grown on a SWNT. a) Reproduced under the terms of the Creative Commons CC‐BY license.^[^ ^180^ ^]^ Copyright 2021, The Authors. Published by Wiley‐VCH. b–d) Reproduced with permission.^[^ ^77^ ^]^ Copyright 2020, American Association for the Advancement of Science.

Besides DWNTs that have been extensively discussed, boron nitride nanotubes,^[^ ^73^, ^74^ ^]^ FNTs,^[^ ^151^, ^152^, ^153^ ^]^ synthetic 1D heterostructures,^[^ ^77^ ^]^ and 2D heterostructures exhibiting anisotropy^[^ ^160^, ^177^, ^178^, ^179^ ^]^ are on the accessible material list of 1D moiré superlattices. The synthetic approach of 1D heterostructures is displayed in **Figure** **7**a.^[^ ^180^ ^]^ So far, synthesis of carbon/hBN, carbon/MoS_2_ (Figure 7b–d) and carbon/hBN/MoS_2_ heteronanotubes have been successfully demonstrated,^[^ ^77^ ^]^ greatly expanding the members of 1D vdW moiré superlattices. It promises the experimental realization of multi‐walled nanotubes composed of two or more tubes of any materials, just like the famous “lego” in 2D.^[^ ^181^ ^]^ But control of atomic registry between adjacent nanotubes is not possible at this point. Also the lengths of heteronanotubes (e.g., carbon/hBN) are limited within few hundreds of nm due to the very slow growth rate of the outer tube via the inner tube template.^[^ ^180^ ^]^ Growing longer heteronanotubes and investigating the growth mechanism are important directions for future research and applications.

Our discussion on the interlayer interactions so far assumes two interacting nanotubes have the same handness (i.e., two nanotubes both either left‐handed or right‐handed). But in reality, the component homo‐ or hetero‐nanotubes can have different handness. This extra degree of freedom in 1D moiré superlattices should be considered and identified in the future experiments to fully resolve the interlayer interactions of 1D moiré superlattices.^[^ ^182^, ^183^ ^]^

The study of 1D vdW moiré superlattices is in its early stage. There are a lot of exciting phenomena to be demonstrated, such as the evidence and signs of moiré flat bands and correlated physics. Meanwhile, experimental challenges on fabricating ultraclean devices and identifying the suitable type of 1D moiré superlattices showing strongly correlated behaviors are waited to be overcome. We envision research attention on 1D vdW moiré superlattices will continue to increase, with substantial amount of efforts and works to be devoted to this emerging field.

## Conflict of Interest

The authors declare no conflict of interest.

## Author Contributions

S.Z., R.K., P.M., M.K., and F.W. contributed equally to this work. All authors participated in researching the data, discussing the content, and contributing to the writing of the manuscript.
